# STEM Neurology & Neuropsychological Research Group Egypt (SNRGE): Localizing Alzheimer's Research in Egypt

**DOI:** 10.1002/alz70861_108902

**Published:** 2025-12-23

**Authors:** Mohamed Khaled Ali Mohamed Ali Zid, Arwa Ayman Nagah Ahmed Shahin, Mohamed Hosni Kamel Elkellawi

**Affiliations:** ^1^ Cairo University Faculty of Medicine, Cairo, Cairo Egypt

## Abstract

**Background:**

The STEM Neurology & Neuropsychological Research Group Egypt (SNRGE) goes on a research into the intricate landscape of Alzheimer's disease (AD), to understand and address the unique challenges of AD in Egypt, SNRGE seeks to redefine the face of Alzheimer's research and treatment. This research transcends laboratory findings, to households and communities in order to reveal the local Alzheimer's discrepancies overwhelmed by the public. By focusing on prevalence studies and local socio‐cultural determinants, SNRGE seeks to strengthen the voice of Alzheimer's affected families to explain the issue in a better image.

**Method:**

SNRGE tasks include conducting comprehensive studies among Egyptian nationals. The studies include the measurement of neuroimaging data, cognition tests, and genetic testing, as well as public health questionnaires. Local hospitals, universities, and national health authorities need to be collaborated with in order to understand the general situation of prevalence and the effect of Alzheimer's in Egypt. In addition, the group has requested to implement screening programs appropriate to Egypt's specific health infrastructure to improve early diagnosis and treatment.

**Result:**

SNRGE’s work has identified several key insights. First, there is a notable underdiagnosis of Alzheimer's in Egypt, often due to cultural and healthcare system barriers. The prevalence of Alzheimer's in Egypt appears to be influenced by both genetic predispositions and environmental factors unique to the region. In addition, SNRGE has found that treatment management in Egypt is significantly affected by societal norms, and there is a lack of a structured support system for families coping with Alzheimer's. This underscores the need for the development of culturally sensitive, community‐based interventions that promote early detection and continuous care.

**Conclusion:**

SNRGE is pioneering Alzheimer's research localization in Egypt, with an emphasis on the cultural, social, and genetic dimensions of the disease. The organization seeks to develop evidence‐based models of treatment that integrate medical science and society's interests. Through connecting clinical research and care delivered at the community level, SNRGE seeks to enhance diagnosis, treatment, and care for Egyptian families suffering from Alzheimer's. Their vision echoes the necessity of localizing neurology research for both scientific necessity and ethical imperative.

